# Suicide under crisis resolution home treatment – a key setting for patient safety

**DOI:** 10.1192/pb.bp.115.051227

**Published:** 2016-08

**Authors:** Isabelle M. Hunt, Louis Appleby, Nav Kapur

**Affiliations:** 1 University of Manchester, Manchester, UK

## Abstract

Recent years have seen a substantial increase in the use of crisis resolution home treatment (CRHT) teams as an alternative to psychiatric in-patient admission. We discuss the functions of these services and their effectiveness. Our research suggests high rates of suicide in patients under CRHT. Specific strategies need to be developed to improve patient safety in this setting.

Crisis resolution home treatment (CRHT) teams were introduced by the National Health Service (NHS) to provide intensive treatment at home for individuals experiencing an acute mental health crisis and who would otherwise be admitted to hospital care. The intended value was for CRHT teams to act as gatekeepers to relieve the pressure on in-patient services, not only through reducing admissions but also by supporting the early discharge of patients from acute wards to home treatment. Following the successful development of crisis intervention models in North America and Australia in the early 1990s, the NHS Plan included a policy mandating the establishment of these teams throughout England.^1^ Targets set within the plan included developing 335 crisis teams by 2004 and delivering home treatment to 100 000 people by 2005. These targets helped bring about a relatively rapid implementation of these services nationwide. They are now available in every trust in England and receive significant NHS investment each year.^2^

Although flexibility exists, the guidelines from the *Mental Health Policy Implementation Guide*^3^ recommended CRHT services for adults aged 18–65 with severe mental illness who would ordinarily require admission to hospital. Typically, CRHT is offered to individuals diagnosed with schizophrenia and affective disorders and excludes those with primary diagnoses of alcohol or drug misuse, personality disorder or intellectual disability. Other key features of CRHT may include 24-hour availability during a crisis, intensive intervention in the early stages of the crisis, and active involvement until the crisis resolves. A core principle of these services is successful engagement with the patient and involvement with the individual's family and social network in the care management plans. Patients are also provided with practical help with social issues, such as financial, housing and childcare arrangements. This holistic approach has contributed to the increase in patient satisfaction associated with receiving care under CRHT,^4^ along with remaining in a familiar environment and the reduction in stigma attached to psychiatric admission.^5,6^ In addition, treatment under CRHT could reduce the need for out-of-area placements – a growing issue within mental health services. Despite these benefits, criticism has been made that CRHT teams are understaffed and patients experience long waiting times before receiving care, often being seen by several different team members.^7^ The gatekeeping role of CRHT services means patients sometimes no longer have the option of choosing to be admitted to hospital, despite some individuals preferring to be treated away from home and the environment that may have triggered the crisis.

The effectiveness of CRHT services has often been examined through assessing their role as gatekeepers and examining changes in admission rates. For example, Jethwa and colleagues^8^ reported a 37.5% reduction in monthly admissions after the introduction of CRHT, whereas Jacobs & Barrenho^9^ found no impact of these services on admission rates. The only UK randomised controlled trial found CRHT users were less likely to be admitted to hospital in the 8 weeks post-crisis compared with the control group receiving standard care from in-patient services and community mental health teams.^10^ However, a national picture of the efficacy of CRHT in terms of admission rates or gatekeeping is difficult to establish with the great variation between healthcare providers in CRHT service delivery.^11^ For example, around a third of CRHT teams do not function as gatekeepers to acute in-patient beds,^11,12^ whereas a report for the National Audit Office found around half of all discharges were not facilitated by CRHT services.^13^

Published evidence on the outcomes of patients under CRHT is limited, particularly with regard to patient safety and serious untoward incidents, including suicide. Studies from the National Confidential Inquiry into Suicide and Homicide by People with Mental Illness (NCISH) have demonstrated that although there has been a significant fall in the number and rate of in-patient suicide over the past decade, there has been a corresponding increase in the number of patient suicide deaths under CRHT.^14^ There are now three times as many suicides under CRHT each year compared with in-patient suicides.^15^ This is not unexpected given the expanding provision of home treatment services and the reduction in hospital admissions. Reassuringly, however, the rate of suicide under CRHT has not increased and has shown a decline between 2003 and 2011.^16^ This would suggest that perhaps some of the safety concerns in CRHTs are being addressed. NCISH findings have also demonstrated that NHS trusts which have introduced 24-hour crisis teams have lower suicide rates compared with areas without these services.^17^

Despite the potential for CRHT to reduce suicide risk, one concern is the possibility that suicide rates are higher in the CRHT setting than the in-patient setting. In our recent study of suicides between 2003 and 2011, we found that the crude rate of suicide among CRHT patients over this period was 14.6 per 10 000 CRHT episodes compared with 8.8 suicides per 10 000 admissions for in-patients.^16^ These rates do not take into account the varying CRHT service models or adjust for case mix or other patient confounders, but reflect overall aggregate national effects. One explanation could be time at risk – if the length of care under CRHT is longer than an in-patient stay this could explain the findings. When we tried to take this into account (by including national estimates of the duration of treatment), the risk under CRHT remained substantially higher than the risk under in-patient care. However, we could not adjust for length of time before and after admission as robust time-at-risk data were not available. The higher rates under CRHT may be indicative of the more intensive treatment and increased staff availability that an admission brings, but could also reflect that the case-loads of CRHT teams increasingly include some of the most acutely ill patients. NCISH findings have shown a high prevalence of known suicide risk factors among patients under CRHT who have subsequently died by suicide, including adverse life events (49%), living alone (44%) or recent discharge from in-patient care (34%).^16^ This raises the question on the suitability of home treatment for vulnerable patients with limited social support or who return to a home environment that has the potential to exacerbate a mental health crisis. We have also recently shown that between 2012 and 2013, 37% of patients who died by suicide under CRHT had been under these services for less than 1 week which may reflect the acuteness and severity of illness.^15^ Changes in service provision may have increased levels of morbidity in admitted and post-discharge populations and the use of CRHT following discharge might mean that some admissions are shortened beyond what is safe. Our previous controlled study on suicide within 2 weeks of discharge found a link between post-discharge deaths and an admission lasting less than 7 days.^18^

What lies ahead for CRHT? Financial pressures could have contributed to a recent trend for trusts to merge their specialist teams, such as CRHT or early intervention with generic community mental health services. However, given the potential benefits of the CRHT model, mental health services should ensure these teams remain a unique treatment option but with effective liaison with in-patient and community mental health services. Variations between providers in CRHT service delivery is of concern and may reflect uncertainties in the evidence base. Guidelines for national standardisation of practice may help to improve the quality of care, especially if these are rooted in sound research. Service developments in crisis home treatment need to be monitored carefully with respect to patient safety to ensure that the right care is being delivered to the right patients.
